# Epigenetic Determinants of *CYP1A1* Induction by the Aryl Hydrocarbon Receptor Agonist 3,3',4,4',5-Pentachlorobiphenyl (PCB 126)

**DOI:** 10.3390/ijms150813916

**Published:** 2014-08-11

**Authors:** Sabine U. Vorrink, Danielle R. Hudachek, Frederick E. Domann

**Affiliations:** 1 Interdisciplinary Graduate Program in Human Toxicology, the University of Iowa, Iowa City, IA 52242, USA; E-Mail: sabine-vorrink@uiowa.edu; 2 Department of Radiation Oncology, the University of Iowa, Iowa City, IA 52242, USA; 3 Summer Undergraduate Research Program, Interdisciplinary Graduate Program in Molecular and Cellular Biology, the University of Iowa, Iowa City, IA 52242, USA; E-Mail: danielle-hudachek@uiowa.edu

**Keywords:** aryl hydrocarbon receptor, chromatin accessibility, DNA methylation, epigenetic regulation, polychlorinated biphenyls

## Abstract

Many enzymes involved in xenobiotic metabolism, including cytochrome P450 (CYP) 1A1, are regulated by the aryl hydrocarbon receptor (AhR). 3,3',4,4',5-Pentachlorobiphenyl (PCB 126) is a potent ligand for AhR and can thus induce the expression of *CYP1A1*. Interestingly, we observed that human carcinoma cell lines derived from different types of epithelial cells displayed divergent degrees of *CYP1A1* induction after exposure to PCB 126. Since epigenetic mechanisms are known to be involved in cell type-specific gene expression, we sought to assess the epigenetic determinants of *CYP1A1* induction in these carcinoma cell lines. In contrast to HepG2 hepatocarcinoma cells, HeLa cervical carcinoma cells showed significantly lower levels of CYP1A1 mRNA expression following PCB 126 exposure. Our results show that the two cell lines maintained differences in the chromatin architecture along the *CYP1A1* promoter region. Furthermore, treatment with the epigenetic modifiers, trichostatin A (TSA) and 5-aza-2'-deoxycytidine (5-Aza-dC), significantly increased the expression of *CYP1A1* after PCB 126 treatment in HeLa cells. However, we did not observe apparent differences in methylation levels or specific location of CpG DNA methylation between the two cell lines in the analyzed *CYP1A1* promoter region. Taken together, our findings suggest that the differences in *CYP1A1* expression between HepG2 and HeLa cells are due to differences in the chromatin architecture of the *CYP1A1* promoter and thus establish a role of epigenetic regulation in cell-specific *CYP1A1* expression.

## 1. Introduction

Humans adapt to exposures to xenobiotics and other chemicals by various mechanisms, one of which is the activation of the aryl hydrocarbon receptor (AhR). The AhR is a transcription factor that mediates the expression of many phase I and phase II drug metabolizing enzymes including the cytochrome P450 (CYP) family, aldehyde dehydrogenase 3 (ALDH3), UDP-glucuronosyl transferase (UGT1A1), and many more [1,2]. Xenobiotic metabolism is a central cellular defense mechanism against foreign substances. An important enzyme that mediates the initial hydroxylation of many chemicals is the monooxygenase cytochrome P450 1A1 (CYP1A1). Cytochrome P450 enzymes, including CYP1A1, are involved in the metabolism of a broad range of chemicals, including polychlorinated biphenyls (PCBs) [2]. In a previous study we showed that exposure to 3,3',4,4',5-pentachlorobiphenyl (PCB 126) resulted in the up-regulation of *CYP1A1* in HepG2 cells [3]. Interestingly, we observed that human cell lines derived from different epithelial cell types varied significantly in their abilities to induce CYP1A1 mRNA expression after PCB 126 exposure.

Epigenetics, the study of heritable changes in gene expression without changes in the genomic DNA sequence, encompasses various regulatory mechanisms such as DNA methylation and histone modifications. Epigenetic regulation plays an important role in genomic imprinting and gene silencing, but perhaps nowhere is it more important than in the establishment and maintenance of different cell types. Additionally, many epigenetic marks are known to change during cancer progression and can affect the expression of oncogenes and tumor suppressor genes [4].

The importance of acetylated histone marks has been assessed in a study by Ovesen *et al.* [5] in which mouse hepatoma-1c1c7 (Hepa-1) cells were treated with various AhR ligands. In contrast to the non-dioxin-like PCB 153 which could not induce changes in histone marks or gene transcription, the dioxin-like and potent AhR ligand PCB 126 recruited the AhR to the *CYP1A1* promoter, increased histone acetylation marks and induced gene transcription. These data show that next to AhR recruitment, changes in histone marks are critical for *CYP1A1* expression [5]. Furthermore, modifications on histone tails can affect the chromatin structure of a gene which in turn can affect the accessibility of various transcription factors and cofactors.

Another important mechanism of epigenetic regulation is DNA methylation. DNA methylation is a chemical modification of the DNA that occurs at the base cytosine to which a methyl group (–CH_3_) is attached. It occurs at CG dinucleotides (called CpGs) which are often enriched in specific regions near gene promoters known as CpG islands. In general, areas of scarce DNA methylation in or around genes are accessible, whereas stretches of very dense methylation, often found in gene promoters, lead to gene silencing [4,6].

Various studies have shown that the chromatin architecture and DNA methylation can affect the regulation of *CYP1A1* gene expression [5,7,8,9]. For example, Okino *et al.* have shown that the *CYP1A1* gene in a human prostate cancer cell line was epigenetically silenced by DNA methylation compared to normal prostate cell lines, and that this methylation was responsible for the lack of *CYP1A1* induction by TCDD [7]. However, whether epigenetic regulation affects *CYP1A1* expression in the context of exposure to PCB 126 has not been analyzed in the human cell lines proposed in this study. Therefore, we sought to assess the epigenetic regulation of *CYP1A1* in two human carcinoma cell lines derived from distinctly different epithelial cell types, HepG2 hepatocarcinoma cells and HeLa cervical carcinoma cells, which showed significantly different levels of *CYP1A1* induction after PCB 126 challenge. We hypothesized that the difference in *CYP1A1* expression between the two cell lines is related to cell type-specific differences in the chromatin structure or DNA methylation profile of the *CYP1A1* promoter. Since epigenetic regulation plays an important role in cell type-specific gene expression, it is important to examine new regulatory mechanisms that affect PCB metabolism and toxicity in different human cell types. This study was designed to determine whether epigenetic regulation plays a role in AhR-mediated induction of *CYP1A1* after exposure to PCB 126 in cell lines derived from different cell types. We found that *CYP1A1* expression was, at least in part, affected by differences in the chromatin structure of the *CYP1A1* promoter, thus supporting a role for epigenetic regulation as a mechanism of differential *CYP1A1* expression in human HepG2 and HeLa cells.

## 2. Results

### 2.1. Differential Inducibility of Cytochrome P450 1A1 (CYP1A1) in HepG2 and HeLa Cells

**Figure 1:**
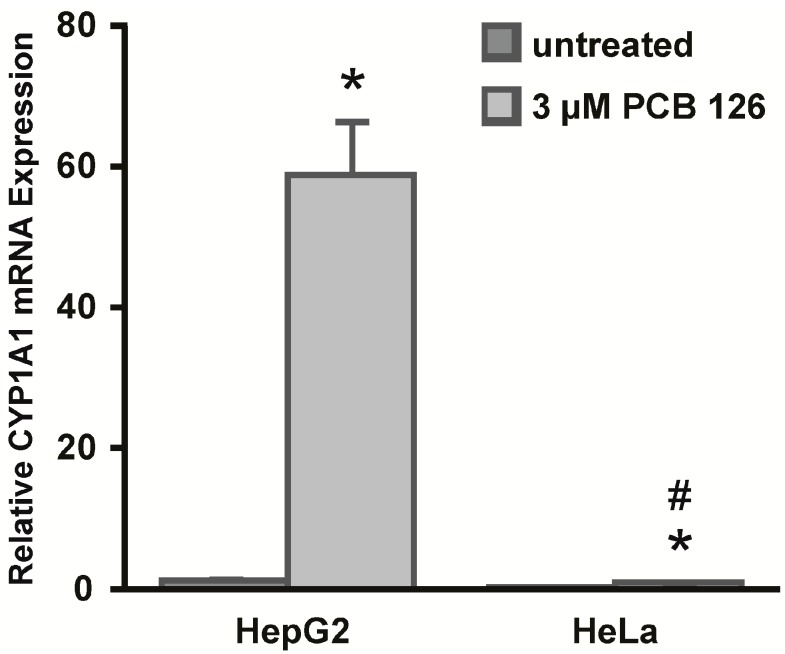
Cytochrome P450 1A1 (CYP1A1) mRNA expression is differentially induced by the aryl hydrocarbon receptor (AhR) agonist 3,3',4,4',5-pentachlorobiphenyl (PCB 126) in HepG2 and HeLa cells. HepG2 and HeLa were treated with 3 µM PCB 126 for 6 h. CYP1A1 mRNA expression was determined by quantitative real-time reverse transcription polymerase chain reaction (qRT-PCR). Analysis was performed with normalization to ribosomal protein, large, P0 (RPLP0) mRNA. RNA expression levels significantly (*p* < 0.05) greater than untreated controls are indicated by an asterisk (*). RNA expression levels significantly (*p* < 0.05) different between PCB 126 treated HepG2 cells and PCB 126 treated HeLa cells are indicated by a number sign (#). Error bars = SEM; *n* = 3.

Two human carcinoma cell lines derived from different types of epithelial cells were analyzed for basal and PCB 126 induced CYP1A1 mRNA expression using qRT-PCR (Figure 1). After treatment with 3 μM PCB 126 for 6 h, HepG2 cells showed significant induction of CYP1A1 mRNA expression compared to untreated controls (58.7 *vs.* 1.1). HeLa cells also induced CYP1A1 mRNA expression but to a significantly lower extent compared to HepG2 cells (0.9 *vs.* 59.7). Furthermore, basal CYP1A1 mRNA levels were nearly eight-fold lower in HeLa cells compared to HepG2 cells (0.15 *vs.* 1.1).

### 2.2. HeLa Cells Show Regions of Open and Closed Chromatin along the CYP1A1 Promoter

**Figure 2:**
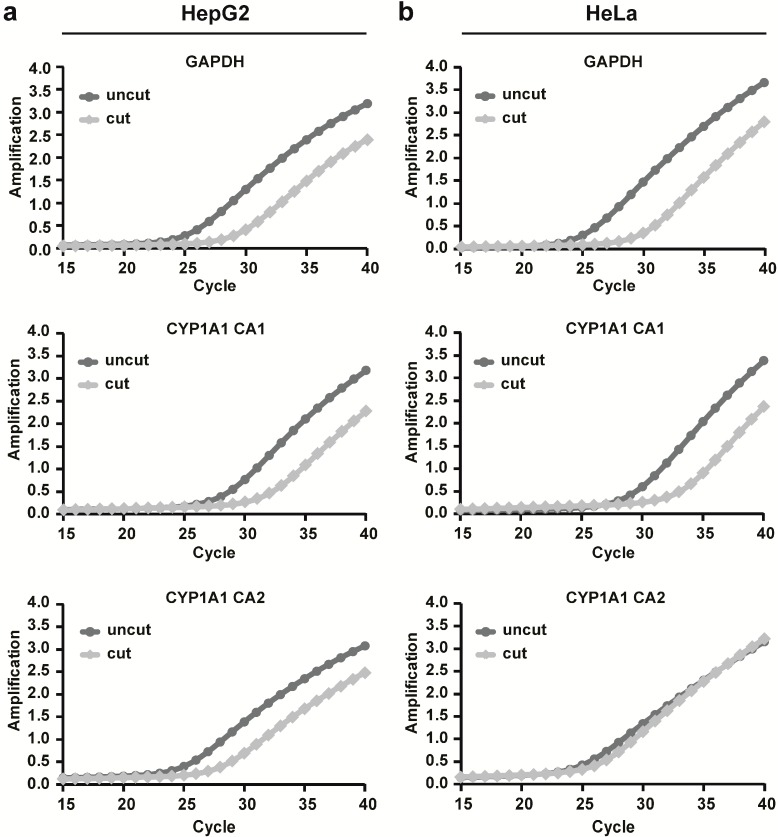
Chromatin accessibility along the *CYP1A1* promoter in HepG2 and HeLa cells. Nuclei of HepG2 cells (**a**) and HeLa cells (**b**) were isolated and digested with DNase. qRT-PCR was used to amplify two different regions of the *CYP1A1* promoter that were also analyzed by bisulfite sequencing. Amplification of *GAPDH* was used as a positive control for accessible chromatin. Results are depicted as magnitude of the signal (∆*R*_n_) *vs.* cycle number.

To analyze whether epigenetic mechanisms of regulation underlie the observed differences in *CYP1A1* expression, chromatin accessibility was measured at two different loci along the *CYP1A1* promoter in HepG2 and HeLa cells. Primer positions are shown in Figure S1. Nuclei of untreated cells were digested with DNase, genomic DNA was isolated and the amplification of two regions along the *CYP1A1* promoter was measured using qRT-PCR (Figure 2). In both cell lines, the amplification curve of the region closest to the transcription start site (CA1) showed a shift to the right (higher *C*_t_) after DNase digestion compared to the undigested control. In contrast, the amplification curve of the region approximately 1.3 kb upstream of the transcription start site (CA2) showed differences between the two cell lines. In HepG2 cells, the amplification curve of the CA2 region showed a shift to the right comparable to the CA1 region. Interestingly, HeLa cells did not show a shift to the right in DNase digested samples but rather amplified similar to the undigested control, thereby suggesting that the chromatin structure is more inaccessible at this region. Together, these data suggest that a difference in the chromatin architecture in the *CYP1A1* 5' regulatory region might play a role in the differential expression of CYP1A1 mRNA in HepG2 and HeLa cells.

### 2.3. 5-Aza-2'-deoxycytidine (5-Aza-dC) Treatment Increases Expression of CYP1A1 mRNA in HeLa Cells

**Figure 3:**
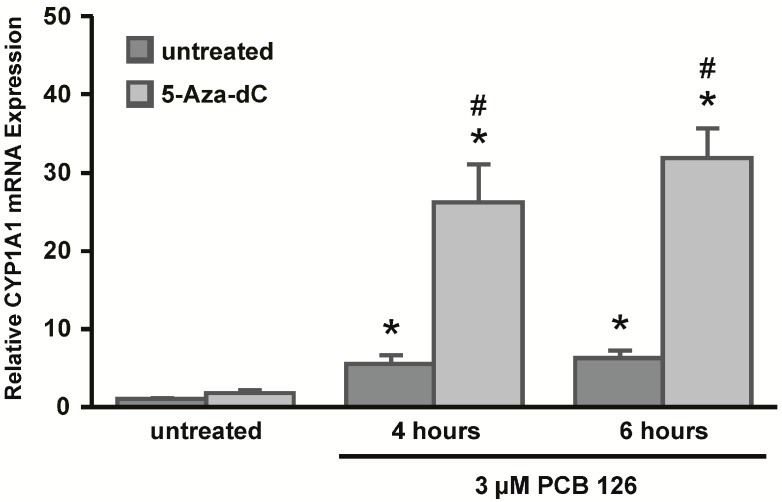
Increased expression of CYP1A1 mRNA triggered by 5-aza-2'-deoxycytidine (5-Aza-dC). HeLa cells were either left untreated or treated with 5-Aza-dC for seven days, and subsequently treated with 3 µM PCB 126 for 4 or 6 h. qRT-PCR was used to assess CYP1A1 mRNA expression with normalization to RPLP0 mRNA. * *p* < 0.05 *vs.* control; ^#^ no drug treatment *vs.* 5-Aza-dC treatment; Error bars = SEM; *n* = 3.

In addition to chromatin structure, the pattern of CpG DNA methylation can play an important role in gene regulation. To test whether CpG DNA methylation plays a role in the expression of *CYP1A1* in HeLa cells, cells were treated with the DNA methyltransferase inhibitor 5-Aza-dC. HeLa cells were cultured in the presence of 5-Aza-dC for seven days prior to exposure to PCB 126. After 6 h of PCB 126 treatment, CYP1A1 mRNA expression was assessed in untreated and 5-Aza-dC-treated cells using qRT-PCR (Figure 3). Consistent with previous findings in this study, HeLa cells showed relatively low induction of CYP1A1 mRNA expression after PCB 126 treatment in the absence of 5-Aza-dC. However, HeLa cells that were treated with 5-Aza-dC prior to PCB 126 exposure showed significantly higher levels of induction of CYP1A1 mRNA expression after cells were treated with PCB 126. These data suggest that DNA methylation might play a role in the regulation and expression of *CYP1A1* in HeLa cells.

### 2.4. HepG2 and HeLa Cells Do not Show Differences in the CpG DNA Methylation Status of the CYP1A1 Promoter

**Figure 4:**
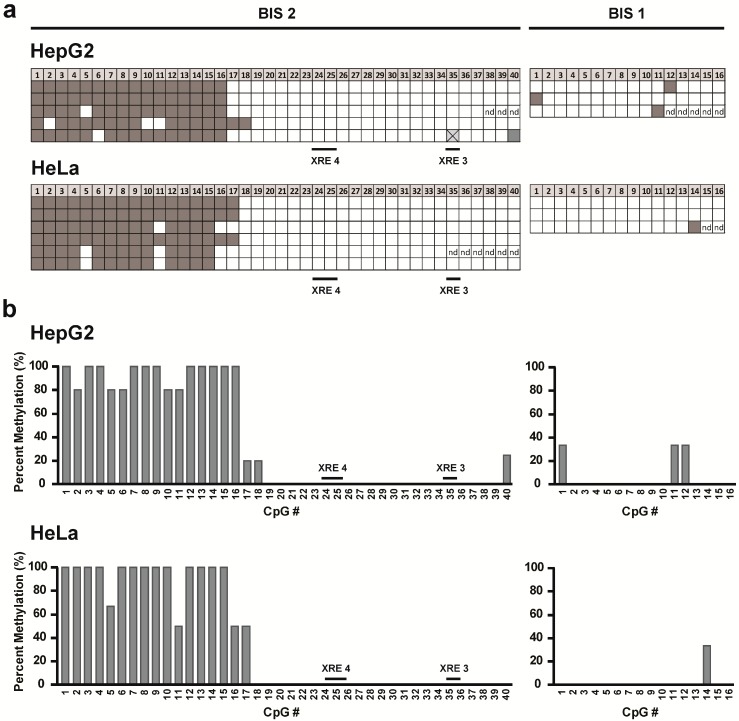
CpG DNA methylation of the *CYP1A1* promoter in HepG2 and HeLa cells. Genomic DNA of HepG2 cells and HeLa cells was bisulfite converted and sequenced. (**a**) Each row of boxes on the grids shows the CpG DNA methylation profile of an individual clone; each box represents a single CpG within the amplified region. The number of analyzed CpG dinucleotides is shown at the top. Grey boxes represent methylated CpG dinucleotides, white boxes represent unmethylated CpG dinucleotides; (**b**) Percent methylation of each individual CpG dinucleotide among the analyzed clones quantified from (**a**).

As we observed an effect of 5-Aza-dC on *CYP1A1* expression, we hypothesized that HepG2 and HeLa cells might show differences in CpG DNA methylation of the *CYP1A1* promoter which might lead to differential *CYP1A1* expression. To assess the CpG DNA methylation status of the *CYP1A1* promoter, primers were designed for two different regions as shown in Figure S1 and CpG DNA methylation was measured using bisulfite sequencing. Figure 4 depicts the methylation status of 56 individual CpG dinucleotides within the amplified regions. Both cell lines showed only one region of accumulation of CpG DNA methylation approximately 1.3 kb upstream of the transcription start site of *CYP1A1*. No methylation was found at the xenobiotic response element (XRE) sequences within the analyzed regions (XRE 3 and XRE 4) in either cell line. Overall, no apparent differences in CpG DNA methylation were found between the two cell lines in the region of the *CYP1A1* promoter analyzed.

### 2.5. Trichostatin A (TSA) Treatment Sensitizes HeLa Cells to PCB 126 Induced CYP1A1 Expression

**Figure 5:**
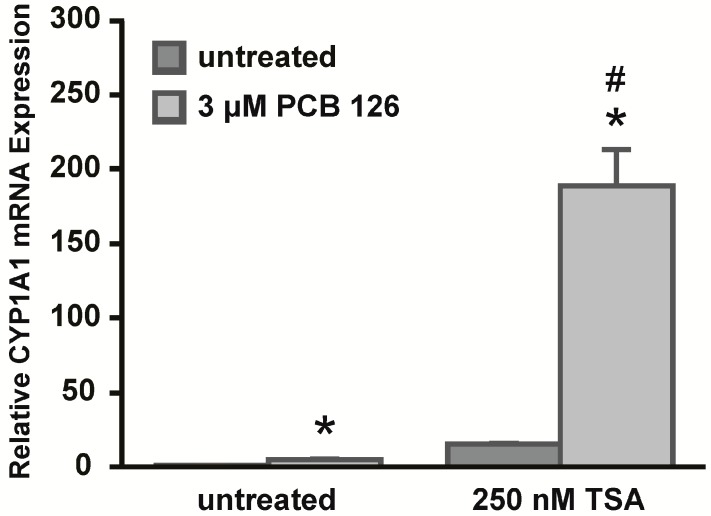
Trichostatin A (TSA) treatment increases CYP1A1 mRNA expression in HeLa cells. HeLa cells were either left untreated or treated with 250 nM TSA for 16 h prior to treatment with 3 µM PCB 126 for 6 h. CYP1A1 mRNA expression was determined by qRT-PCR with normalization to RPLP0 mRNA. * *p* < 0.05 *vs.* control; ^#^ no drug treatment *vs.* TSA treatment; Error bars = SEM; *n* = 3.

Because our early results showed differences in the chromatin architecture between HepG2 and HeLa cells, we sought to analyze whether treatment with the histone deacetylase (HDAC) inhibitor trichostatin A (TSA) would affect the expression of *CYP1A1* in HeLa cells. HeLa cells were left untreated or treated with TSA prior to treatment with PCB 126 as described in materials and methods (Figure 5). PCB 126 treatment induced *CYP1A1* expression at significantly higher levels in TSA-treated cells compared to non-TSA-treated cells. Furthermore, basal levels of *CYP1A1* expression were higher in TSA-treated cells compared to non-TSA-treated cells. Overall, these data support that the chromatin architecture and its associated histone acetylation state are important for the regulation and expression of *CYP1A1* in HeLa cells.

### 2.6. HeLa Cells Express High Basal Levels of Aryl Hydrocarbon Receptor Repressor (AhRR) mRNA

**Figure 6:**
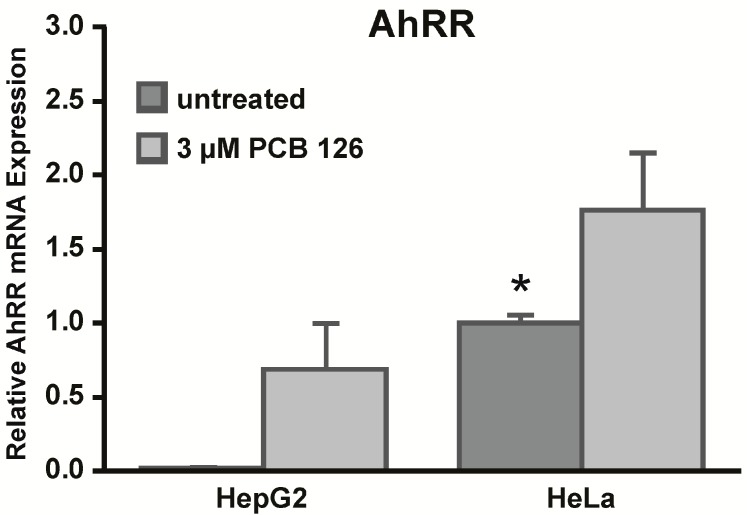
Aryl hydrocarbon receptor repressor (AhRR) mRNA expression in HepG2 and HeLa cells. HepG2 and HeLa cells were treated with 3 µM PCB 126 for 6 h and AhRR mRNA expression levels were determined using qRT-PCR. Analysis was performed with normalization to RPLP0. * *p* < 0.05 HeLa *vs.* HepG2; Error bars = SEM; *n* = 3.

**Figure 7:**
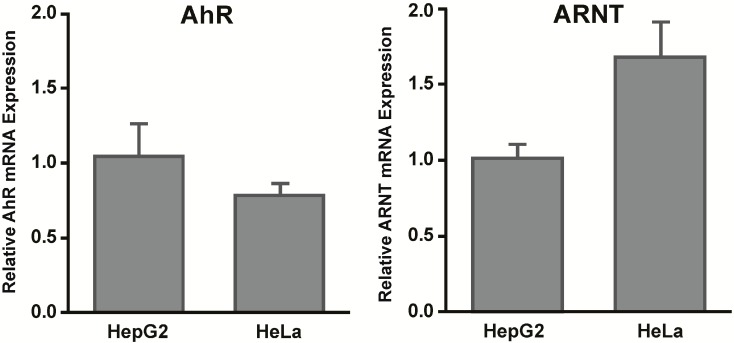
Relative aryl hydrocarbon receptor (AhR) and aryl hydrocarbon receptor nuclear translocator (ARNT) mRNA levels in HepG2 and HeLa cells. Basal AhR and ARNT mRNA expression levels were measured in untreated HepG2 and HeLa cells using qRT-PCR. Analysis was performed with normalization to RPLP0. Error bars = SEM; *n* = 3.

To assess regulatory mechanisms other than epigenetic regulation that might be involved in the expression of *CYP1A1*, we measured mRNA expression levels of the aryl hydrocarbon receptor repressor (AhRR) (Figure 6). We measured basal and PCB 126 induced AhRR mRNA expression in HepG2 and HeLa cells and found that both cell lines increased the expression of AhRR mRNA after PCB 126 exposure. Importantly, HeLa cells expressed significantly higher basal levels of AhRR compared to HepG2 cells which might also contribute to the lower responses to PCB 126 in HeLa cells. Next to the AhRR, the relative amounts of AhR and/or ARNT might play a role in gene regulation as well. However, when we assessed basal AhR and ARNT levels, we did not observe apparent differences in the expression of these genes between the cell lines (Figure 7).

## 3. Discussion

Xenobiotic metabolism includes a multitude of enzymes and can be divided into two parts: phase I and phase II metabolism. Enzymes involved in phase I metabolism aid in the initial metabolism of foreign compounds making them more suitable substrates for subsequent metabolic and detoxifying steps [2]. However, this initial step can also lead to bioactivation and the formation of reactive metabolites [10]. Phase II enzymes are involved in the subsequent conjugation of the compounds generated in phase I metabolism. An important family of enzymes involved in phase I drug metabolism is the cytochrome P450 family; the expression of which is regulated by various receptors including the AhR which induces the expression of the CYP1 family of enzymes. Various chemicals, including PCB 126, are known to induce the AhR and thus CYP1 expression.

Numerous studies have shown that the chromatin structure and DNA methylation profile of *CYP1A1* can play a role in *CYP1A1* expression in cells treated with AhR ligands [5,7,8,9]. In this study, we observed that two human carcinoma cell lines derived from different types of epithelial cells showed varying intensities of CYP1A1 mRNA expression after exposure to PCB 126. We hypothesized that cell type-specific differences in the epigenetic regulation of the *CYP1A1* promoter might play a role in *CYP1A1* expression in PCB 126 exposed human cells. Thus, we sought to assess the chromatin structure and CpG DNA methylation profile of *CYP1A1* in both HepG2 and HeLa cells.

First, we assessed chromatin accessibility to protein:DNA binding around the *CYP1A1* promoter in HepG2 and HeLa cells using a nuclease accessibility assay. HepG2 and HeLa cells were chosen to assess cell-specific differences in *CYP1A1* expression because they represent different epithelial cell types of origin and they respond differentially to PCB 126 induced CYP1A1 mRNA expression. Interestingly, both cell lines showed an open chromatin conformation in the CA1 region of the *CYP1A1* promoter. However, we observed differences between the two cell lines in the CA2 promoter region. In contrast to HepG2 cells, HeLa cells did not show a shift of the amplification curve of this region which suggests the presence of a more closed chromatin structure at this region in these cells. Interestingly, both regions assessed for chromatin accessibility contain a XRE sequence (CA1 contains XRE 1 and CA2 contains XRE 5). These results are consistent with findings reported in the ENCODE (Encyclopedia of DNA Elements) database for HepG2 and HeLa cells, which show several regions of differential chromatin accessibility spanning the *CYP1A1* gene 5'-end and HepG2 with a generally more open and active promoter region than HeLa. Taken together, the observed difference in chromatin accessibility, particularly at XRE 5, might be a reason why we observed differences in *CYP1A1* expression between the two cell lines.

Next, we asked whether differences in CpG DNA methylation, which can affect the accessibility of chromatin, might be associated with the observed differences in chromatin accessibility. Methyl-binding proteins recognize methylated DNA and can subsequently recruit histone-modifying enzymes that in turn affect the chromatin structure at the site of CpG methylation [6]. Initially we treated HeLa cells, which showed significantly lower levels of *CYP1A1* induction after PCB 126 exposure compared to HepG2 cells and which showed regions of closed chromatin along the *CYP1A1* promoter, with the DNA methyltransferase inhibitor 5-Aza-dC. Continued exposure to this drug leads to a reduction in overall DNA methylation as cells proliferate and cells might thus re-express previously methylated genes. We hypothesized that if CpG DNA methylation of the *CYP1A1* promoter plays a role in gene regulation, CYP1A1 mRNA expression could be increased after drug treatment. Indeed, after seven days of 5-Aza-dC treatment, HeLa cells significantly increased CYP1A1 mRNA expression after PCB 126 challenge. HeLa cells that were not treated with 5-Aza-dC induced *CYP1A1* to a much lower extent. These data suggest that DNA methylation might play a role in *CYP1A1* gene expression in HeLa cells. However, because 5-Aza-dC is an inhibitor of DNA methyltransferases, the reduction in CpG DNA methylation is not specific to a particular locus within the genome, but rather CpG DNA methylation gets lost across the whole genome. To specifically assess the CpG methylation status of the *CYP1A1* promoter and to compare the methylation pattern between HepG2 and HeLa cells, we used bisulfite sequencing. Genomic DNA was treated with sodium bisulfite to convert unmethylated cytosines to uracil. Methylated cytosines are not affected by this process and thus remain unchanged. Therefore, any CpG dinucleotides retained in the final sequence were originally methylated. Our sequencing data showed that only a few of the 56 CpG dinucleotides analyzed were methylated. Interestingly, the genomic region in which we found CpG DNA methylation corresponds to the region in which we observed differences in chromatin accessibility. However, when we compared the two cell lines, we observed no apparent differences in the amount and location of CpG DNA methylation. Our findings are entirely consistent with data in the ENCODE database where CpG methylation was measured by reduced representation bisulfite sequencing (RRBS) as well as the Illumina Infinium HumanMethylation450 BeadChip array in several cell types including HepG2 and HeLa. Finally, the promoter region in which two XRE sequences are located (XRE 3 and XRE 4) showed no CpG DNA methylation in either cell line.

Although we did not observe apparent differences in CpG DNA methylation in the regions we analyzed, our data suggest that the chromatin structure seems to be a major regulator of *CYP1A1* expression. To further investigate the role of the chromatin architecture, experiments were performed with the HDAC inhibitor TSA. Acetylated histones are associated with an open chromatin structure and transcriptionally active chromatin, thus inhibiting HDAC enzymes might lead to a more accessible chromatin structure and hence gene expression. Chromatin accessibility experiments in HeLa cells suggested a more closed chromatin structure of *CYP1A1*, thus we hypothesized that treatment with TSA might increase *CYP1A1* expression in response to PCB 126. Indeed, HeLa cells treated with TSA prior to PCB 126 treatment showed significantly higher levels of *CYP1A1* expression compared to their non-TSA-treated counterparts. In summary, the results of this study suggest that the chromatin architecture, associated with a state of acetylated histones, appears to be a major regulator of *CYP1A1* expression and thus plays an important role in the epigenetic regulation of this gene. Differences in histone states across different cell types are also evident in the ENCODE database and therefore support this conclusion. Therefore, in this study we have revealed a new mechanism of epigenetic regulation of *CYP1A1* expression in human carcinoma cell lines derived from different types of epithelial cells.

Although our data do not specifically show that the *CYP1A1* promoter itself is epigenetically silenced by CpG DNA methylation, our 5-Aza-dC results suggest that CpG DNA methylation might still play a role in the regulation of *CYP1A1*. A diverse set of proteins is involved in the regulation of *CYP1A1* gene expression including the AhR, ARNT, p300/CBP (a histone acetyltransferase), Sp1 and TFIIB (both transcription factors), and many other cofactors [1]. Each of these proteins can be affected by epigenetic regulation and thus might be silenced by CpG DNA methylation. The lack of other transcription factors or cofactors might also affect the expression of *CYP1A1* even though the *CYP1A1* promoter is intact and functional. Another protein that plays an important role in the expression of AhR-regulated target genes is the aryl hydrocarbon receptor repressor (AhRR). The AhRR is a target gene of the AhR and functions as a negative feedback regulator by competing with the AhR for ARNT [11,12]. Furthermore, the AhRR has been shown to bind to XRE sites of *CYP1A1* and thus can mediate nonresponsiveness to AhR ligands in certain cells [13]. We measured *AhRR* mRNA expression in both cell lines and found that HeLa cells expressed significantly higher basal levels of AhRR compared to HepG2 cells. Higher baseline AhRR expression might be an explanation why we observed lower responses to PCB 126 in HeLa cells. Importantly, our results confirm and extend those of an earlier study by Tsuchiya *et al.* who observed high constitutive AhRR mRNA expression in HeLa cells [14]. Furthermore, we did not observe differences in basal mRNA expression levels of AhR or ARNT in HepG2 and HeLa cells, findings that support results by Iwanari *et al.* who similarly detected no differences in AhR and ARNT mRNA expression between HepG2, HeLa, and other cell lines [15].

## 4. Materials and Methods

### 4.1. Chemicals and Polychlorinated Biphenyl (PCB) Treatment

H.J. Lehmler of the Iowa Superfund Research Program at the University of Iowa (Iowa City, IA, USA) kindly provided us with 3,3',4,4',5-pentachlorobiphenyl (PCB 126). PCB stock solutions were prepared in dimethyl sulfoxide (DMSO) and added to the cell culture medium. PCB 126 exposures were done in serum-free medium at a concentration of 3 μM. This concentration was chosen based on the observation that human blood serum levels of PCBs in a population in Alabama, USA range from 0.003 to 6.5 μM [16]. 5-Aza-2'-deoxycytidine (5-Aza-dC) was purchased from Sigma (St. Louis, MO, USA) and stock solutions were prepared in water at the time of exposure. Trichostatin A (TSA) was purchased as a stock solution in DMSO from Sigma and was diluted in serum-free medium prior to exposure. Cell lines were left untreated as control cultures.

### 4.2. Cell Lines and Cell Culture Conditions

Human hepatocellular carcinoma (HepG2) cells were purchased from the American Type Culture Collection (ATCC) (Manassas, VA, USA). Human cervical adenocarcinoma (HeLa) cells, originally purchased from ATCC as WRL68 cells [17], were a kind gift from Dr. Kyle Brown at the Iowa City Veterans Affairs Medical Center, Iowa City, IA, USA. These cell lines were chosen because they showed differential responses to PCB 126 induced CYP1A1 mRNA expression. HepG2 cells were grown in Dulbecco’s Modified Eagle Medium (DMEM) supplemented with 10% fetal bovine serum (FBS) and penicillin-streptomycin. HeLa cells were grown in Minimum Essential Medium (MEM) supplemented with 10% FBS, 1 mM sodium pyruvate, 0.15% sodium bicarbonate, 2 mM glutamine, non-essential amino acids, and penicillin-streptomycin. All cell lines were maintained in a humidified 37 °C incubator with 5% CO_2_ and routinely subcultured before reaching confluence by washing and detaching cells with TrypLE Express.

### 4.3. Quantitative Real-Time RT-PCR

mRNA expression levels were assessed by harvesting total RNA with TRIzol reagent (Invitrogen, Grand Island, NY, USA). An amount of 500 ng of total RNA was reverse transcribed for 2 h at 37 °C using the High Capacity cDNA Archive Kit (Applied Biosystems, Foster City, CA, USA). For quantitative real-time RT-PCR (qRT-PCR) analysis the Universal ProbeLibrary (UPL) system (Roche Applied Science, Indianapolis, IN, USA) was used. Primers and probes were designed using the online Universal ProbeLibrary Assay Design Center. AhR, AhRR, ARNT, CYP1A1, and ribosomal protein, large, P0 (RPLP0) mRNA expression was measured using the following primers and probes: AhR forward primer: 5'-AGCCGGTGCAGAAAACAG-3', reverse primer: 5'-CTATGCCGCTTGGAAGGAT-3', UPL probe: #33; AhRR forward primer: 5'-TGCTTCATCTGCCGTGTG-3', reverse primer: 5'-AGCTGCCAAGCCTGTGAC-3', UPL probe: #56; ARNT forward primer: 5'-CTACCCGCTCAGGCTTTTC-3', reverse primer: 5'-CACCAAACTGGGAAGTACGAG-3', UPL probe: #3; CYP1A1 forward primer: 5'-GGTCAAGGAGCACTACAAAACC-3', reverse primer: 5'-TGGACATTGGCGTTCTCAT-3', UPL probe: #2; RPLP0 forward primer: 5'-TCTACAACCCTGAAGTGCTTGAT-3', reverse primer: 5'-CAATCTGCAGACAGACACTGG-3', UPL probe: #6. For each qRT-PCR reaction (10 µL reaction volume), TaqMan Universal PCR Master Mix (Applied Biosystems) was used along with primer pairs at 0.5 µM and UPL probes at 0.2 µM. Of the prepared cDNA, 10 ng was used for each qRT-PCR reaction. The PCR protocol was as follows: DNA polymerase heat-activation at 95 °C for 10 min followed by 40 cycles, denaturing at 95 °C for 15 s, annealing and elongating at 60 °C for 1 min. The ABI PRISM 7500 sequence detection system (Applied Biosystems) was used for data collection. The cycle threshold (*C*_t_) for each sample was calculated by selecting an amplification threshold in the linear range of each sample. The relative mRNA levels were calculated as follows: Δ*C*_t_ (sample) = *C*_t_ (mRNA of interest) − *C*_t_ (RPLP0); ΔΔ*C*_t_ = Δ*C*_t_ (treatment) − Δ*C*_t_ (control); relative expression = 2^−ΔΔ*C*^^t^.

### 4.4. Isolation of Nuclear Extracts and Chromatin Accessibility

Nearly confluent cells were washed with 1× PBS, harvested with 1× PBS containing 1 mM PMSF, and collected by centrifugation. After the pellet was resuspended in 5× packed cell volume equivalent of hypotonic lysis buffer (10 mM Tris–HCl, pH 7.5, 2 mM MgCl_2_, 3 mM CaCl_2_, and 0.32 M sucrose) supplemented with 10 mM NaF, 1 mM NaVO_4_, 1 mM PMSF, and 1× protease inhibitor cocktail (Roche Applied Science; Complete Mini), the pellet was incubated for 15 min on ice to allow the cells to swell. The cells were then lysed by the addition of 0.87% NP-40 and vortexed. After centrifugation, the supernatant was collected (cytoplasmic extract). The remaining nuclear pellet was washed with hypotonic lysis buffer containing 1 mM PMSF and 0.8% NP-40 and centrifuged. The washed nuclei were resuspended in 400 μL digestion buffer (50 mM Tris pH 7.5, 100 mM NaCl, 10 mM MgCl_2_, and 1 mM DTT) containing 20 μg RNaseA. One hundred μL of the digestion mixture were left untreated or were enzymatically digested with 10 units RQ1 DNase (Promega, Madison, WI, USA) for 10 min at 37 °C. DNA of undigested and digested samples was isolated using the DNeasy Blood and Tissue Kit (Qiagen, Valencia, CA, USA). Of the isolated DNA, 20 ng was used as a template for each qRT-PCR (10 μL reaction volume). Primers were designed to two regions along the *CYP1A1* promoter as shown in Figure S1. CA1: forward primer: 5'-GCGCGAACCTCAGCTAGT-3', reverse primer: 5'-TTCCCGGGGTTACTGAGTC-3', UPL probe: #23; CA2: forward primer: 5'-AGCTAGGCACGCAAATACAAC-3', reverse primer: 5'-CTGTGGCACGACACGAAG-3', UPL probe: #27; GAPDH: 5'-CCTTCCGTGCAGAAACCTC-3', reverse primer: 5'-CTGGCTCCTGGCATCTCT-3', UPL probe: #12. GAPDH chromatin accessibility was measured as a positive control.

### 4.5. 5-Aza-dC and TSA Treatment

For 5-Aza-dC experiments, HeLa cells were seeded on day one in two 10 cm dishes (650,000 cells/dish). On day two, one dish was treated with 5 μM freshly prepared 5-Aza-dC (Sigma) in DMEM medium containing 10% FBS for a total of seven days. The control dish was left untreated. Medium was removed and fresh 5-Aza-dC was added on day four. On day six, cells were split into 60 mm dishes (250,000 cells/dish) in DMEM medium containing 10% FBS and 5-Aza-dC. The next day, the medium was removed and serum-free DMEM supplemented with 5-Aza-dC was added to the cells. Cells were allowed to incubate in serum-free medium for 8 h. On day eight, the cells were treated with 3 μM PCB 126 for 4 or 6 h and total RNA was extracted using TRIzol reagent (Invitrogen). For TSA experiments, HeLa cells were treated with 250 nM TSA (Sigma) for 16 h in serum-free DMEM medium prior to treating cells with 3 μM PCB 126 for 6 h and total RNA was extracted using TRIzol reagent (Invitrogen).

### 4.6. Bisulfite Sequencing

Genomic DNA was extracted from cell lines using the DNeasy Tissue Kit (Qiagen, Valencia, CA, USA). A total of 1 μg of DNA was bisulfite converted using the EZ DNA Methylation Kit (Zymo Research Corporation, Irvine, CA, USA). Four micro liters of bisulfite-converted DNA was amplified in a nested PCR reaction using HotStarTaq Master Mix (Qiagen) and primer pairs at 0.5 μM. Primers were designed to amplify the *CYP1A1* promoter of bisulfite-converted DNA but not unmodified DNA. For primer design, the MethPrimer online tool was used [18]. For the outer PCR (10 μL reaction volume), the following primers were used: Bis_out forward primer: 5'-TTTTTGGAAATTTTGTAATAGGAAG-3', Bis_out reverse primer: 5'-AAAAACCCACTATAAAACACATCTC-3' (1193 bp). The reaction was heated to 95 °C for 10 min followed by 40 cycles, denaturing at 95 °C for 1 min, annealing at 52 °C for 1 min and elongating at 72 °C for 1.5 min followed by a final elongation step at 72 °C for 5 min. For the nested inner PCR (25 μL reaction volume), two different primer sets were used Bis_1 forward primer: 5'-TTTGTGTTTTGTTAATTAAAGTATTAGTTA-3', Bis_1 reverse primer: 5'-TAAAAACACCAAAAATCCCAATTC-3' (577 bp); Bis_2 forward primer: 5'-TGGAAATTTTGTAATAGGAAGGTTT-3', Bis_2 reverse primer: 5'-TTTAATTAACAAAACACAAAAATCC-3' (580 bp). The reaction was amplified as above but annealing was carried out at 51 °C and elongation was carried out at 72 °C for 1 min. After the final PCR, the reactions were run on an agarose gel and the correct bands were excised and extracted from the gel using the Qiagen Gel Extraction Kit. The purified PCR products were cloned into the pJET1.2 vector using the CloneJet PCR Cloning Kit (Thermo Scientific, Waltham, MA, USA), and the ligation mixtures were used for transformation of competent *E. coli* DH5α. Plasmids were isolated from overnight cultures using the PureLink Quick Plasmid Miniprep Kit (Invitrogen). Correct plasmids were identified by digestion with FastDigest *BglII* in FastDigest *BglII* Green Buffer (Thermo Scientific) for 20 min at 37 °C. Plasmids with the correct insert were sequenced at the University of Iowa DNA Sequencing Facility, and the methylation status of the available CpG dinucleotides in the PCR products was assessed.

### 4.7. Statistical Analysis

Groups of data were analyzed using a *t*-test to determine significant differences between groups. Statistical probability of *p* < 0.05 was considered significant. Three independent biological replicates (*n* = 3) were used for each dataset unless otherwise noted.

## 5. Conclusions

The induction of drug metabolizing enzymes after exposure to foreign chemicals plays a significant role in cellular defense mechanisms against these substances. However, the expression of drug metabolizing enzymes can be affected by epigenetic regulatory mechanisms including the chromatin conformation, CpG DNA methylation, and histone acetylation. In this study we used two human carcinoma cell lines derived from different human epithelial cell types, HepG2 and HeLa, to assess cell type-specific differences in *CYP1A1* expression. The results of this study show that the two cell lines were differentially sensitive to CYP1A1 mRNA induction after PCB 126 exposure. Our data suggest that differences in chromatin accessibility of the *CYP1A1* promoter in a region rich in AhR binding sites provides a new regulatory mechanism for differential CYP1A1 mRNA expression in these cell lines. Therefore, this study establishes a role of epigenetic regulation in the expression of *CYP1A1*.

## Supplementary Files

Supplementary File 1

## Abbreviations

5-Aza-dC, 5-aza-2'-deoxycytidine; AhR, aryl hydrocarbon receptor; AhRR, aryl hydrocarbon receptor repressor; ARNT, aryl hydrocarbon receptor nuclear translocator; CYP1A1, cytochrome P450 1A1; DMSO, dimethyl sulfoxide; HDAC, histone deacetylase; PCB, polychlorinated biphenyl; PCB 126, 3,3',4,4',5-pentachlorobiphenyl; qRT-PCR, quantitative real-time reverse transcription polymerase chain reaction; RPLP0, ribosomal protein, large, P0; TSA, trichostatin A; XRE, xenobiotic response element.
