# Supplementary material for: Epigenetic Determinants of CYP1A1 Induction by the Aryl Hydrocarbon Receptor Agonist 3,3',4,4',5-Pentachlorobiphenyl (PCB 126)
Source: Int J Mol Sci. 2014 Aug 11;15(8):13916–31. doi: 10.3390/ijms150813916 (PMC4159831; doi:10.3390/ijms150813916)

Supplementary Information

Figure S1. Schematic representation of the human cytochrome P450 1A1 (*CYP1A1)* 5' regulatory region. Triangles show the location of xenobiotic response element (XRE) sequences in the promoter region. The transcription start site of *CYP1A1* is represented as a bent arrow. The locations of the promoter regions analyzed by bisulfite sequencing (BIS 1 and BIS 2) and chromatin accessibility (CA1 and CA2) are indicated.

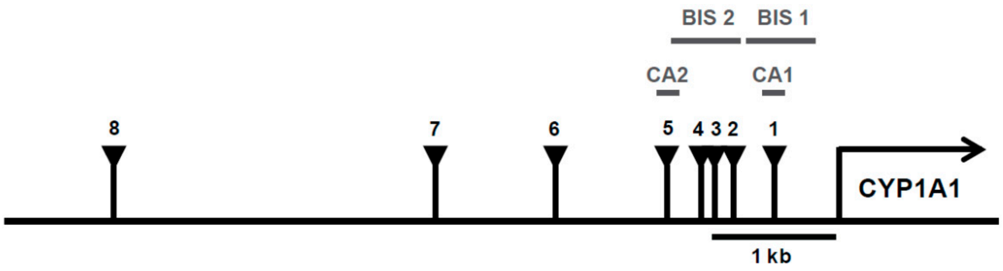

Supplement: Supplementary File 1 [file ijms-15-13916-s001.pdf]
